# Knockdown of long non-coding RNA SOX21-AS1 attenuates amyloid-β-induced neuronal damage by sponging miR-107

**DOI:** 10.1042/BSR20194295

**Published:** 2020-03-27

**Authors:** Wanru Xu, Kai Li, Qian Fan, Biyun Zong, Ling Han

**Affiliations:** Internal Medicine-Neurology, Heze Municipal Hospital, Heze, Shandong, China

**Keywords:** Alzheimer's disease, amyloid-β, jiangcan1996930@126.com, miR-107

## Abstract

**Background:** Alzheimer’s disease (AD), which has no effective drugs to delay or prevent its progression, is a multifactorial complex neurodegenerative disease. Long non-coding RNA SOX21 antisense RNA1 (SOX21-AS1) is associated with the development of AD, but the underlying molecular mechanism of SOX21-AS1 in AD is still largely unclear.

**Methods:** To construct the AD model, SH-SY5Y and SK-N-SH cells were treated with amyloid-β_1-42_ (Aβ_1-42_). Quantitative real-time polymerase chain reaction (qRT-PCR) was executed to detect the expression of SOX21-AS1 and miRNA-107. Western blot analysis was utilized to assess the levels of phosphorylated Tau (p-Tau). 3-(4,5-dimethylthiazol-2-yl)-2,5-diphenyltetrazolium bromide (MTT) or flow cytometry assay was employed to determine the viability and apoptosis of SH-SY5Y and SK-N-SH cells. The relationship between SOX21-AS1 and miRNA-107 was verified with the dual-luciferase reporter assay.

**Results:** SOX21-AS1 expression was augmented while miR-107 expression was decreased in Aβ_1-42_-treated SH-SY5Y and SK-N-SH cells. Moreover, Aβ_1-42_ elevated the levels of p-Tau and impeded viability and induced apoptosis of SH-SY5Y and SK-N-SH cells. Also, SOX21-AS1 silencing attenuated Aβ_1-42_ mediated the levels of p-Tau, viability, and apoptosis of SH-SY5Y and SK-N-SH cells. Importantly, SOX21-AS1 acted as a sponge for miR-107 in SH-SY5Y and SK-N-SH cells. Furthermore, the increase in p-Tau levels and apoptosis and the repression of viability of Aβ_1-42_-treated SH-SY5Y and SK-N-SH cells mediated by miR-107 inhibition were partly recovered by SOX21-AS1 depletion.

**Conclusion:** SOX21-AS1 silencing could attenuate Aβ_1-42_-induced neuronal damage by sponging miR-107, which provided a possible strategy for the treatment of AD.

## Introduction

Alzheimer’s disease (AD) is a complex neurodegenerative disorder characterized by memory loss, cognitive and functional abilities decline that can lead to dementia [1]. The emphatic pathological features of AD are extracellular amyloid-β peptide (Aβ) deposition and intracellular neurofibrillary tangles formation [2]. Aβ is a neurotoxic agent that can couple with neurons and trigger cell death under certain aggregation conditions [3]. However, Aβ is a product of metabolism in the body, and its excessive deposition and clearance imbalance can lead to the occurrence of AD [4]. Neurofibrillary tangles are composed of highly p-Tau protein, and its accumulation is deemed to be closely connected to cognitive recession of AD [5]. At present, there are no effective treatments to delay or prevent the development of AD. Therefore, it is essential to explore the mechanism of the occurrence and development of AD to develop effective AD treatment strategies.

Long non-coding RNA (lncRNAs) are class of transcripts over 200 nucleotides in length that cannot be translated into proteins and exert a vital role in human physiology and disease processes [6]. There have been reported to be involved in transmission of genetic information, such as transcription factor recruitment, alternative splicing control, and mRNA stability and translational activity [7]. A substantial amount of evidence indicated that lncRNAs were involved in pluripotency maintenance, euro-ectodermal differentiation, epigenetic imprinted neuron-specific relaxation, brain tissue patterning and synaptogenesis, and mechanisms of epigenetic underlie memory formation [8]. Long non-coding RNA SOX21 antisense RNA1 (SOX21-AS1) is a recently discovered lncRNA, located on chromosome 13q32.1, and transcribed into a transcript of 2986 nucleotides [9]. Recent literature had revealed that SOX21-AS1 could reduce oxidative stress and inhibit neuronal apoptosis in AD mice [10]. However, the underlying molecular mechanisms of SOX21-AS1 in the pathogenesis of AD are rarely reported.

MicroRNAs (miRNAs) is a class of non-coding RNAs with approximately 21 nucleotides, which can be used as an important molecular tool for human disease diagnosis and prognosis evaluation [11]. They could regulate a variety of biological processes, containing neuronal differentiation, regeneration and survival [12]. Studies had shown that a large amount of miRNAs were involved in the progression of neurodegenerative disorder [13,14]. MicroRNA-107 (miR-107) was reported to be down-regulated in AD [15]. To date, it is unclear whether miR-107 is regulated by SOX21-AS1 and participates in the process of AD.

In the present study, we probed into the role of SOX21-AS1 in the pathogenesis of AD and its underlying molecular mechanism by constructing an AD cell model, and provided a possible strategy for the prevention and treatment of AD.

## Materials and methods

### Cell culture and treatment

SH-SY5Y and SK-N-SH cells were obtained from the American Type Culture Collection (ATCC, Manassas, VA, U.S.A.). All cells were cultured in Dulbecco’s modified Eagle’s medium (DMEM) supplemented with 10% fetal bovine serum (Gibco, Thermo Fisher Scientific, Waltham, MA, U.S.A.). The above cells were maintained in an incubator in a humidified with 5% CO_2_ at 37°C.

Aβ_1-42_ was purchased from Sigma-Aldrich (St. Louis, MO, U.S.A.) for the construction of the AD cell model. First, in order to aggregate Aβ_1-42_, 0.5 mg Aβ_1-42_ was dissolved in double distilled water at 37°C for 7 d. Then, the thioflavin T assay was used to assess the aggregation of Aβ_1-42_. Next, the aggregation of Aβ_1-42_ was diluted to a concentration of 200 μM and stored at −80°C. SH-SY5Y and SK-N-SH cells were treated with different concentrations of Aβ_1-42_ (0, 5, 10, 20 μM) for 24 h or Aβ_1-42_ (10 μM) for different times (0, 12, 24, 48 h).

### Cell transfection

Small interference RNA (siRNA) targeting SOX21-AS1 (si-SOX21-AS1) and negative control (si-NC), pcDNA-SOX21-AS1 overexpression vectors (SOX21-AS1) and pcDNA were obtained from Genepharma (Shanghai, China). The miRNA mimic and inhibitor targeting miR-107 (miR-107 and anti-miR-107) and their corresponding negative controls (miR-NC or anti-miR-NC) were obtained from RIBOBIO (Guangzhou, China). Oligonucleotides or plasmids were transfected into SH-SY5Y and SK-N-SH cells using Lipofectamine 2000 reagent (Invitrogen, Carlsbad, CA, U.S.A.) according to the instructions of manufacturer.

### Quantitative real-time polymerase chain reaction (qRT-PCR)

Total RNA of SH-SY5Y and SK-N-SH cells with or without Aβ_1-42_ treatment was extracted using Trizol reagent (Invitrogen). The High-Capacity complementary DNA (cDNA) Reverse Transcription Kits (Thermo Fisher Scientific) or MicroRNA Reverse Transcription Kits (Thermo Fisher Scientific) was used to generate cDNA for SOX21-AS1 and miR-107. The SYBR® Premix DimerEraser Kit (TaKaRa, Dalian, China) was applied to analyze the levels of SOX21-AS1 and miR-107. The relative expression of SOX21-AS1 and miR-107 was figured by the 2^−ΔΔCt^ method, and glyceraldehyde-3-phosphate dehydrogenase (GAPDH) or U6 small nuclear RNA (snRNA) was served as an internal control. The primers for amplification were exhibited as below: SOX21-AS1 (forward, 5ʹ-AGCTACGGAGGAAGAGGGTT-3 ʹ; reverse, 5ʹ-TCAGCAGCGCATGTAAGTGA-3ʹ), GAPDH (forward, 5ʹ-GACTCCACTCACGGCAAATTCA-3ʹ; reverse, 5ʹ-TCGCTCCTGGAAGATGGTGAT-3ʹ), miR-107 (forward, 5ʹ-AGCAGCATTGTACAGGGCTATCA-3ʹ; reverse, 5ʹ-TCGCTCCTGGAAGATGGTGAT-3ʹ) and U6 snRNA (forward, 5ʹ-GCTCGCTTCGGCAGCACA-3ʹ; reverse, 5ʹ-GAGGTATTCGCACCAGAGGA-3ʹ).

### Cell viability assay

The viability of SH-SY5Y and SK-N-SH cells with or without Aβ_1-42_ treatment was detected by the 3-(4,5-dimethylthiazol-2-yl)-2,5-diphenyltetrazolium bromide (MTT) kit (Promega Corporation, Madison, WI, U.S.A.). Briefly, SH-SY5Y and SK-N-SH cells (5Cor^4^ cells/well) were treated with different concentrations of Aβ_1-42_ (0, 5, 10, 20 μM) for 24 h or Aβ_1-42_ (10 μM) for different times (0, 12, 24, 48 h). Subsequently, MTT (20 μl, 5 mg/ml) was added to each well and maintained for 4 h. Next, dimethyl sulfoxide solution was added to dissolve the formazan crystals. The optical density (OD) at 490 nm was measured using the Microplate Reader (Thermo Fisher Scientific).

### Flow cytometry assay

The apoptosis of SH-SY5Y and SK-N-SH cells with or without Aβ_1-42_ treatment were detected by an annexin V-FITC assay kits (Invitrogen). In brief, treated cells were harvested and re-suspended with binding buffer (1×). Then, annexin V-FITC (5 μl) and propidium iodide (10 μl) were added to the binding buffer and incubated for 20 min in the dark. The apoptotic rate was determined by a FACScan® flow cytometry (BD Biosciences, San Jose, CA, U.S.A.).

### Western blot assay

Total protein of SH-SY5Y and SK-N-SH cells with or without Aβ_1-42_ treatment was extracted with RIPA lysis buffer (Beyotime, Shanghai, China). BCA assay kit (Pierce, Rockford, IL, U.S.A.) was used to detect the protein concentration. Total protein were separated by electrophoresis on 8−12% sodium dodecyl sulphate-polyacrylamide gel electrophoresis (SDS-PAGE) and then transferred onto polyvinylidene difluoride (PVDF) membranes (Millipore, Billerica, MA, U.S.A.). After that, TBST buffer with 5% skim milk was used to block the PVDF membrane for 2 h at 37°C. Then, the PVDF membranes were incubated with primary antibodies: rabbit anti-p-Tau (ab109309, 1:10000, Abcam, Cambridge, MA, U.S.A.) and rabbit-anti-β-actin (ab179467, 1:5000, Abcam) at 4°C overnight. Next, the PVDF membrane was washed and then incubated with goat anti-rabbit IgG (ab6721, 1:2000, Abcam) antibody for 1 h at 37°C. β-Actin was regarded as a loading control. The bands were visualized through the Immobilon™ Western Chemiluminescent HRP Substrate (Millipore).

### Dual-luciferase reporter assay

The binding sites between SOX21-AS1 and miR-107 were predicted with LncBase v.2 database. The sequences of SOX21-AS1 (with predicted miR-107 binding sites) and mutant SOX21-AS1 were cloned into the pGL3-control luciferase reporter vectors (Promega, Madison, WI, U.S.A.) for the construction of the luciferase reporter vectors SOX21-AS1-WT and SOX21-AS1-MUT. Then, the luciferase reporter vectors were co-transfected with miR-107 or miR-NC into SH-SY5Y and SK-N-SH cells using Lipofectamine 2000 transfection reagent (Invitrogen), respectively. The luciferase activity of the luciferase reporter vectors was assessed using dual-luciferase Reporter assay kit (Promega).

### RNA immunoprecipitation (RIP) assay

EZ-Magna RIP RNA-Binding Protein Immunoprecipitation Kit (Millipore) was utilized to verify the relationship between SOX21-AS1 and miR-107 based on the manufacture’s protocol. Briefly, cells were transfected with miR-107 or miR-NC for 48 h and then lysed in RIP buffer. Subsequently, the lysates of cells were maintained in a RIP buffer containing magnetic beads conjugated that are coupled with Ago2 or IgG antibody (Millipore). The enrichment of SOX21-AS1 was assessed by qRT-PCR.

### Statistical analysis

The data in the study were derived from at least three independent experiments. Statistical analysis was conducted by SPSS 17.0 (SPSS, Chicago, IL, U.S.A.), and *P* < 0.05 was deemed to indicate a statistically significant difference. The differences between two or among more groups in the present study were determined using Student’s *t* test or one-way analysis of variance followed by Tukey post-hoc test, respectively. Data from repeated experiments were presented as mean ± standard deviation.

## Results

### SOX21-AS1 was up-regulated in Aβ_1-42_-treated SH-SY5Y and SK-N-SH cells

**Figure 1:**
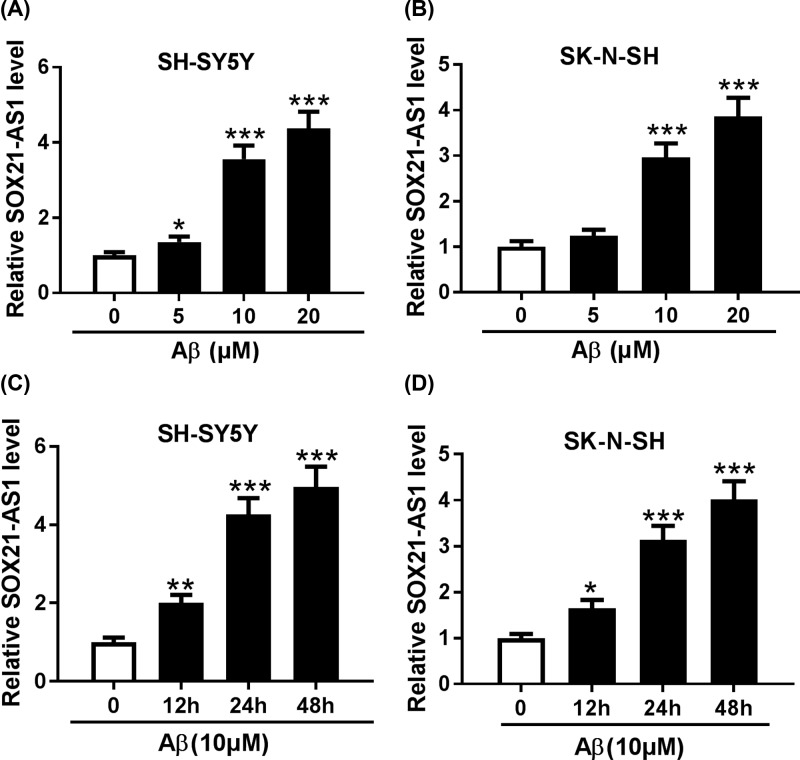
Expression levels of SOX21-AS1 in Aβ_1-42_-treated SH-SY5Y and SK-N-SH cells (**A** and **B**) QRT-PCR was utilized to detect SOX21-AS1 expression in SH-SY5Y (A) and SK-N-SH (B) cells treated with different concentrations of h Aβ_1-42_. (**C** and **D**) QRT-PCR was used to determine SOX21-AS1 expression in Aβ_1-42_-treated (10 μM) SH-SY5Y (C) and SK-N-SH (D). GAPDH was used as an internal control for SOX21-AS1. *P <0.05, **P <0.01 and ***P <0.001.

In the first place, we assessed the expression of SOX21-AS1 in both SH-SY5Y and SK-N-SH cells treated with different concentrations of Aβ_1-42_ (0, 5, 10, 20 μM) using qRT-PCR. The results showed that SOX21-AS1 expression was prominently elevated in SH-SY5Y and SK-N-SH cells treated with 10 and 20 μM Aβ_1-42_ (Figure 1A,B). Subsequently, the expression of SOX21-AS1 in SH-SY5Y and SK-N-SH cells treated with Aβ_1-42_ (10 μM) for different times (0, 12, 24, 48 h) was evaluated via qRT-PCR. We discovered that SOX21-AS1 was obviously up-regulated in Aβ_1-42_-treated SH-SY5Y and SK-N-SH cells in a time-dependent manner (Figure 1C,D). Therefore, these results hinted that increased SOX21-AS1 expression might be connected with the pathogenesis of AD.

### Knockdown of SOX21-AS1 attenuated Aβ_1-42_ mediated viability and apoptosis of SH-SY5Y and SK-N-SH cells

**Figure 2:**
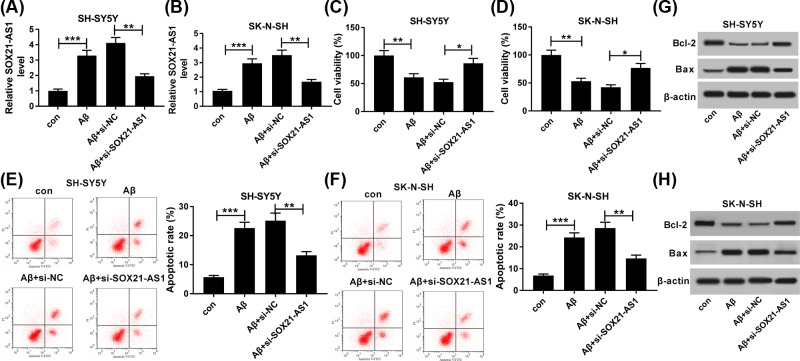
Knockdown of SOX21-AS1 attenuated Aβ-induced cell viability and apoptosis in SH-SY5Y and SK-N-SH cells Aβ_1-42_-treated (10 μM) SH-SY5Y and SK-N-SH cells were transfected with si-NC or si-SOX21-AS1. (**A** and **B**) The expression of SOX21-AS1 in Aβ_1-42_-treated SH-SY5Y (A) and SK-N-SH (B) cells was detected by using qRT-PCR. (**C** and** D**) The viability of Aβ_1-42_-treated SH-SY5Y (C) and SK-N-SH (D) cells was determined by MTT assay. (**E** and **F**) The apoptosis of Aβ_1-42_-treated SH-SY5Y (E) and SK-N-SH (F) cells was detected by flow cytometry. (**G** and **H**) The expression of Bcl-2 and Bax proteins in Aβ_1-42_-treated SH-SY5Y (G) and SK-N-SH (H) cells was examined with western blot analysis. *P <0.05, **P <0.01 and ***P <0.001.

In consideration of the above findings, SH-SY5Y and SK-N-SH cells treated with Aβ_1-42_ (10 μM) for 24 h were selected for further studies. First, Aβ_1-42_-treated SH-SY5Y and SK-N-SH cells were transfected with si-NC or si-SOX21-AS1 to silence the expression of SOX21-AS1. As shown in Figure 2A,B, the enhancement of SOX21-AS1 in SH-SY5Y and SK-N-SH cells induced by Aβ_1-42_ was partly restored by the knockdown of SOX21-AS1, indicating that the si-SOX21-AS1 could be used for subsequent analysis. Afterward, the effects of SOX21-AS1 suppression on the viability and apoptosis of Aβ_1-42_-treated SH-SY5Y and SK-N-SH cells were determined by MTT or flow cytometry assays. Results of the MTT assay indicated that the inhibition of viability of SH-SY5Y and SK-N-SH cells induced by Aβ_1-42_ was partially reversed by SOX21-AS1 silencing (Figure 2C,D). Besides, flow cytometry assay revealed that SOX21-AS1 knockdown partially restored the facilitation of apoptosis of SH-SY5Y and SK-N-SH cells mediated by Aβ_1-42_ (Figure 2E,F). Also, Aβ_1-42_ constrained the expression of Bcl-2 protein and elevated the expression of Bax in SH-SY5Y and SK-N-SH cells, while this influence was overturned by SOX21-AS1 suppression (Figure 2G,H). Taken together, knockdown of SOX21-AS1 could recover the viability and apoptosis of SH-SY5Y and SK-N-SH cells induced by Aβ_1-42_.

### SOX21-AS1 inhibition overturned Aβ_1-42_ mediated the levels of p-Tau of SH-SY5Y and SK-N-SH cells

**Figure 3:**
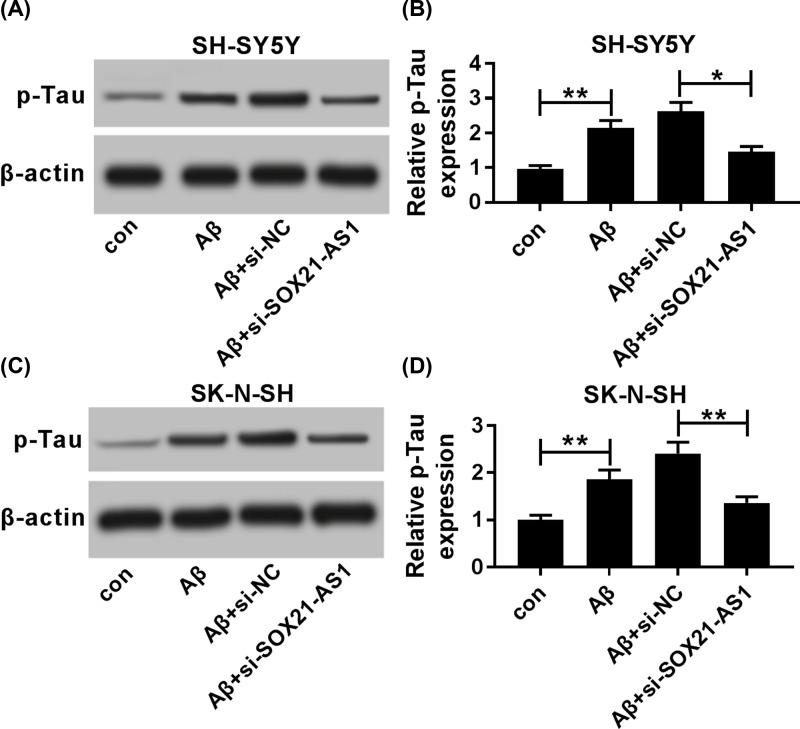
Effect of SOX21-AS1 knockdown on p-Tau levels of Aβ-induced SH-SY5Y and SK-N-SH cells Aβ_1-42_-treated SH-SY5Y and SK-N-SH cells were transfected with si-NC or si-SOX21-AS1. (**A−D**) Western blot analysis was performed to examine the levels of p-Tau in Aβ_1-42_-treated SH-SY5Y (A and B) and SK-N-SH (**C,D**) cells; *P <0.05 and **P <0.01.

Subsequently, the influence of SOX21-AS1 silencing on the levels of p-Tau of Aβ_1-42_-treated SH-SY5Y and SK-N-SH cells was assessed by Western blot analysis. The results presented that Aβ_1-42_ remarkably increased the level of p-Tau in SH-SY5Y and SK-N-SH cells, while this enhancement was overturned by the introduction of si-SOX21-AS1 (Figure 3A–D). These findings indicated that SOX21-AS1 down-regulation could undermine Aβ_1-42_ mediated the levels of p-Tau in SH-SY5Y and SK-N-SH cells.

### SOX21-AS1 served as a sponge for miR-107 in SH-SY5Y and SK-N-SH cells

**Figure 4:**
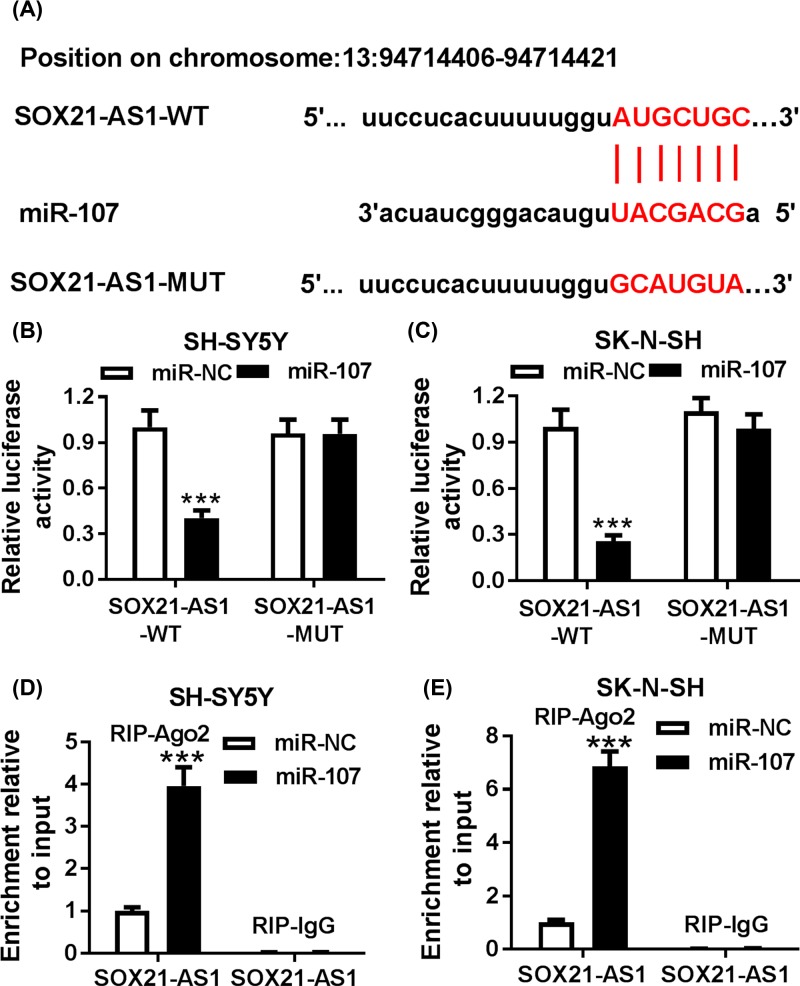
SOX21-AS1 served as a sponge for miR-107 in SH-SY5Y and SK-N-SH cells (**A**) LncBase v.2 database was used for the prediction of the binding sites between SOX21-AS1 and miR-107. (**B** and **C**) The luciferase activity of the reporter vectors SOX21-AS1-WT or SOX21-AS1-MUT in SH-SY5Y (B) and SK-N-SH (C) cells transfected with miR-NC or miR-107 was determined by dual-luciferase reporter assay. (**D** and **E**) RIP assay was performed for the analysis of the relationship between SOX21-AS1 and miR-107 in SH-SY5Y (D) and SK-N-SH (E) cells. U6 snRNA was served as an internal control for miR-107; ***P <0.001.

To further explore the underlying molecular mechanism of SOX21-AS1 in pathogenesis of AD, the LncBase v.2 database was utilized for the prediction of the potential target of SOX21-AS1. Bioinformatics analysis presented that miR-107 possessed a complementary base-paring region with SOX21-AS1 (Figure 4A). To verify this prediction, the luciferase vectors SOX21-AS1-WT and SOX21-AS1-MUT were constructed. Results of dual-luciferase reporter assay displayed that the luciferase activity of the luciferase reporter vectors SOX21-AS1-WT in SH-SY5Y and SK-N-SH cells transfected with miR-107 was remarkably reduced than the control group, whereas the luciferase activity of the luciferase reporter vectors SOX21-AS1-MUT did not changed (Figure 4B,C). Then, RIP assay was performed for further analysis of the relationship between SOX21-AS1 and miR-107. The results indicated that the enrichment of SOX21-AS1 in the Ago2-immunoprecipitation complex in SH-SY5Y and SK-N-SH cells transfected with miR-107 was markedly higher than that in the miR-NC group (Figure 4D,E). In sum, these results manifested that SOX21-AS1 acted as a sponge for miR-107 in SH-SY5Y and SK-N-SH cells.

### MiR-107 was negatively regulated by SOX21-AS1 in Aβ_1-42_-treated SH-SY5Y and SK-N-SH cells

**Figure 5:**
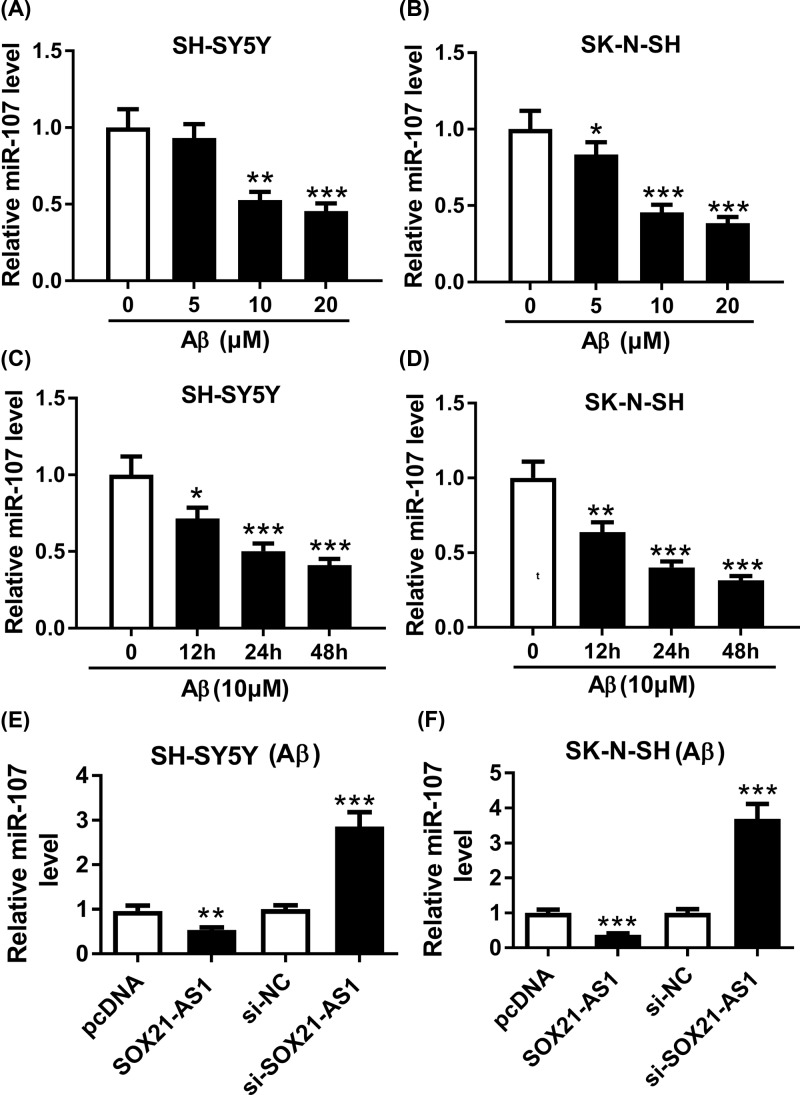
MiR-107 was negatively regulated by SOX21-AS1 (**A** and** B**) The expression of miR-107 was detected using qRT-PCR in SH-SY5Y (A) and SK-N-SH (B) cells treated with different concentrations of Aβ_1-42_. (**C** and** D**) The expression of miR-107 was measured by using qRT-PCR in Aβ_1-42_-treated (10 μM) SH-SY5Y (C) and SK-N-SH (D) cells. (**E** and **F**) The expression of miR-107 in Aβ_1-42_-treated SH-SY5Y (E) and SK-N-SH (F) transfected with pcDNA, SOX21-AS1, si-NC or si-SOX21-AS1 was determined through using qRT-PCR; *P <0.05, **P <0.01 and ***P <0.001.

In view of the above findings, we examined the expression of miR-107 in SH-SY5Y and SK-N-SH cells treated with different concentrations of Aβ_1-42_ (0, 5, 10, 20 μM) for 24 h (Figure 5A,B). We found that miR-107 was dramatically downregulated in SH-SY5Y and SK-N-SH cells treated with Aβ_1-42_ (10 μM) for different times (0, 12, 24, 48 h) through qRT-PCR. The results exhibited that miR-107 expression was obviously reduced in Aβ_1-42_-treated SH-SY5Y and SK-N-SH cells in a time-dependent manner (Figure 5C,D). Also, we found that the expression of miR-107 was drastically inhibited in Aβ_1-42_-treated SH-SY5Y and SK-N-SH cells transfected with SOX21-AS1 than the pcDNA vectors (Figure 5E,F). Moreover, the expression of miR-107 was strikingly elevated in Aβ_1-42_-treated SH-SY5Y and SK-N-SH cells transfected with si-SOX21-AS1 in comparison with the control group (Figure 5E,F). Together, these data revealed that miR-107 was negatively modulated by SOX21-AS1 in Aβ_1-42_-treated SH-SY5Y and SK-N-SH cells.

### Knockdown of SOX21-AS1 reversed miR-107 inhibition-induced viability and apoptosis of Aβ_1-42_-treated SH-SY5Y and SK-N-SH cells

**Figure 6:**
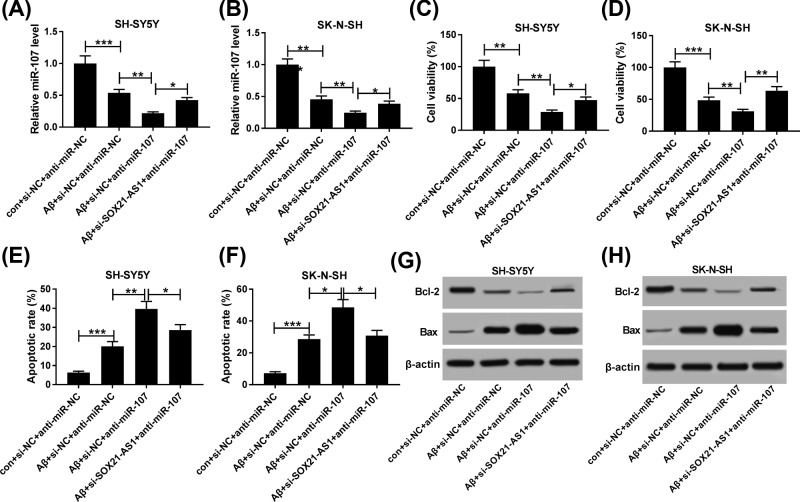
Knockdown of SOX21-AS1 reversed miR-107 inhibition-induced viability and apoptosis of Aβ_1-42_-treated SH-SY5Y and SK-N-SH cells (**A–F**) SH-SY5Y and SK-N-SH cells with or without Aβ_1-42_ treatment were transfected with si-NC+anti-miR-NC, si-NC+anti-miR-107 or si-SOX21-AS1+anti-miR-107. (**A** and **B**) The expression of miR-107 was detected via qRT-PCR in SH-SY5Y (**A**) and SK-N-SH **(B**) cells. (**C** and** D**) MTT assay was utilized to detect the viability of SH-SY5Y (**C**) and SK-N-SH (**D**) cells. (E and F) Flow cytometry assay was performed for the detection of the apoptosis of SH-SY5Y (**E**) and SK-N-SH (**F**) cells. (**G** and **H**) Protein levels of Bcl-2 and Bax in SH-SY5Y (**G**) and SK-N-SH (**H**) cells were evaluated with Western blot analysis; *P <0.05, **P <0.01 and ***P <0.001.

Next, we further investigated whether SOX21-AS1 affects the viability and apoptosis through miR-107 in Aβ_1-42_-treated SH-SY5Y and SK-N-SH cells. As exhibited in Figure 6A,B, miR-107 expression was dramatically decreased in Aβ_1-42_-treated SH-SY5Y and SK-N-SH cells transfected with anti-miR-107 compared with the anti-miR-NC group, while this reduction was reversed by SOX21-AS1 inhibition. MTT assay showed that the repression of viability of Aβ_1-42_-treated SH-SY5Y and SK-N-SH cells caused by miR-107 inhibition was abolished by the knockdown of SOX21-AS1 (Figure 6C,D). In addition, flow cytometry assay revealed that inhibition of miR-107 markedly accelerated the apoptosis of Aβ_1-42_-treated SH-SY5Y and SK-N-SH cells, while this effect was abolished by SOX21-AS1 suppression (Figure 6E,F). Furthermore, both the inhibition of Bcl-2 protein and the acceleration of Bax protein in Aβ_1-42_-treated SH-SY5Y and SK-N-SH cells mediated by miR-107 silencing were restored by SOX21-AS1 depletion (Figure 6G,H). Therefore, these results indicated that SOX21-AS1 mediated the viability and apoptosis of Aβ_1-42_-treated SH-SY5Y and SK-N-SH cells via miR-107.

### SOX21-AS1 repression weakened miR-107 inhibition-induced the levels of p-Tau of Aβ_1-42_-treated SH-SY5Y and SK-N-SH cells

**Figure 7:**
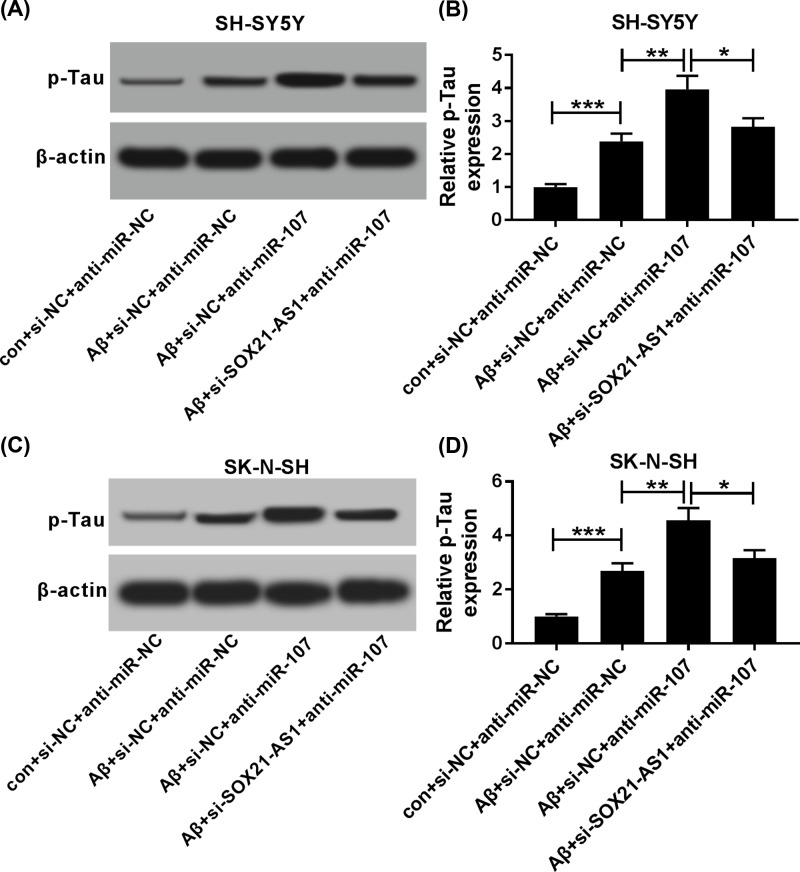
Influence of SOX21-AS1 knockdown on miR-107 inhibition-mediated p-Tau levels of Aβ_1-42_-treated SH-SY5Y and SK-N-SH cells (**A–D**) SH-SY5Y and SK-N-SH cells with or without Aβ_1-42_ treatment were transfected with si-NC+anti-miR-NC, si-NC+anti-miR-107, or si-SOX21-AS1+anti-miR-107. (A**–**D) The levels of p-Tau in SH-SY5Y (A and B) and SK-N-SH (C and D) cells were assessed by Western blot analysis; *P <0.05, **P <0.01 and ***P <0.001.

Considering that the above findings, we further probed whether SOX21-AS1 affects the levels of p-Tau by miR-107 in Aβ_1-42_-treated SH-SY5Y and SK-N-SH cells. Western blot analysis manifested that silenced miR-107 expression could markedly elevate the level of p-Tau in Aβ_1-42_-treated SH-SY5Y and SK-N-SH cells, while this increase was recovered by SOX21-AS1 knockdown (Figure 7A**–**D). Collectively, these data disclosed that SOX21-AS1 regulated the levels of p-Tau by miR-107 in Aβ_1-42_-treated SH-SY5Y and SK-N-SH cells.

## Discussion

AD is a neurodegenerative disease associated with the elderly, whose pathology shows obvious deposition of Aβ and formation of neurofibrillary tangles. Aβ can self-aggregate to form oligomers which are toxic to the neuronal cells [16]. Studies had shown that Aβ induced neurotoxicity similar to the pathology of AD, and was widely used to construct AD model *in vitro* [17]. It was reported that lncRNAs were associated with neurological diseases, such as neurodegenerative disorder and epilepsy [18,19]. Studies had revealed that the hyper-phosphorylation of Tau leads to the formation of neurofibrillary tangles, which is highly related to the neurodegenerative changes in AD [20,21]. In the present study, we constructed the AD cell model *in vitro* to explore the role and potential molecular mechanism of SOX21-AS1 in the pathogenesis of AD.

Previous studies pointed out that lncRNAs exist curial roles in the pathogenesis of AD. For instance, knockdown of lncRNA BACE1-AS could enhance memory and learning behaviors in AD animal model [22]. Zhao et al. disclosed that lncRNA NEAT1 promoted the progression of AD by modulating the miR-124/BACE1 axis [23]. Also, reduced lncRNA EBF3-AS expression could overturn Aβ-induced apoptosis of SH-SY5Y cells [24]. In the present study, Aβ could enhance p-Tau protein level, repressed viability and promoted apoptosis of SH-SY5Y and SK-N-SH cells. Also, SOX21-AS1 was up-regulated in Aβ_1-42_-treated SH-SY5Y and SK-N-SH cells. Moreover, SOX21-AS1 inhibition could restore Aβ-mediated p-Tau levels, viability, and apoptosis of SH-SY5Y and SK-N-SH cells. Report of Zhang et al. reported that SOX21-AS1 silencing could suppress apoptosis of hippocampal neuron cells and enhance memory and learning ability in AD mice [10]. These findings manifested that SOX21-AS1 acted as a pathogenic factor in AD, and which was in line with the report of Zhang et al.

LncRNA had been shown to be involved in gene regulation as sponges for miRNAs or as endogenous competitive RNA [25]. Hu et al. suggested that miR-107 down-regulation could repress SYK expression and inactivate the NF-KB signaling pathway, which promoted the deterioration of spatial memory [26]. A previous report revealed that miR-107 family silencing might be played a vital role in AD pathogenesis via enhancing CDK5 activity [27]. Shu et al. claimed that miR-107 mimics overturned the enhancement of p-Tau induced by Aβ in mice [28]. In the present study, we found that SOX21-AS1 served as a sponge for miR-107 in SH-SY5Y and SK-N-SH cells. Besides, miR-107 was decreased in Aβ_1-42_-treated SH-SY5Y and SK-N-SH cells, and SOX21-AS1 repression reversed miR-107 inhibition-mediated p-Tau levels, viability, and apoptosis of Aβ_1-42_-treated SH-SY5Y and SK-N-SH cells. These results concluded that SOX21-AS1 mediated Aβ-induced neuronal damage via miR-107.

In conclusion, SOX21-AS1 was up-regulated while miR-107 was down-regulated in Aβ_1-42_-treated SH-SY5Y and SK-N-SH cells. Knockdown of SOX21-AS1 could attenuate Aβ_1-42_ mediated p-Tau level, viability, and apoptosis of SH-SY5Y and SK-N-SH cells. In addition, SOX21-AS1 acted as a sponge for miR-107 in SH-SY5Y and SK-N-SH cells. Also, SOX21-AS1 knockdown reversed miR-107 inhibition-mediated p-Tau levels, viability, and apoptosis of Aβ_1-42_-treated SH-SY5Y and SK-N-SH cells. Therefore, we concluded that SOX21-AS1 inhibition attenuated Aβ_1-42_-induced neuronal damage through sponging miR-107, which provided a possible strategy for the treatment of AD.

## Highlights

1. Aβ_1-42_ increased the levels of p-Tau and suppressed viability and promoted apoptosis of SH-SY5Y and SK-N-SH cells.
2. SOX21-AS1 was augmented in Aβ_1-42_-treated SH-SY5Y and SK-N-SH cells, and SOX21-AS1 down-regulation reversed Aβ_1-42_ mediated the levels of p-Tau, cell viability and apoptosis in SH-SY5Y and SK-N-SH cells.
3. SOX21-AS1 acted as a sponge for miR-107 in SH-SY5Y and SK-N-SH cells.
4. MiR-107 expression was decreased in Aβ_1-42_-treated SH-SY5Y and SK-N-SH cells, and SOX21-AS1 knockdown overturned the effects of miR-107 inhibition on p-Tau levels, viability, and apoptosis of Aβ_1-42_-treated SH-SY5Y and SK-N-SH cells.

## Abbreviations

Aβ_1-42_: amyloid-β_1-42_
AD: Alzheimer’s disease
MTT: 3-(4,5-dimethylthiazol-2-yl)-2,5-diphenyltetrazolium bromide
p-Tau: phosphorylated Tau
qRT-PCR: quantitative real-time polymerase chain reaction
